# Chinese medicinal formula Fufang Xueshuantong capsule could inhibit the activity of angiotensin converting enzyme

**DOI:** 10.1080/13102818.2014.911611

**Published:** 2014-07-18

**Authors:** Shujing Sheng, Yonggang Wang, Chaofeng Long, Weiwei Su, Xia Rong

**Affiliations:** ^a^ Guangzhou Quality R&D Center of Traditional Chinese Medicine, Guangdong Key Laboratory of Plant Resources, School of Life Sciences, Sun Yat-sen University, Guangzhou, PR China; ^b^ Guangdong Zhongsheng Pharmaceutical Co., Ltd., Dongguan, PR China; ^c^ Guangzhou Blood Center, Guangzhou, 510095, China

**Keywords:** Fufang Xueshuantong capsule, angiotensin converting enzyme, *Panax notoginseng*, saponins

## Abstract

Fufang Xueshuantong (FXST) capsule, a Chinese medicinal formula composed of four herbals – *Panax notoginseng*, *Radix Astragali*, *Radix Salvia Miltiorrhizae* and *Radix Scrophulariaceae*, has been used to treat cardiovascular diseases for many years, but the pharmacological mechanisms underlying its effects has not been clarified. This study investigates if a connection between FXST and angiotensin converting enzyme (ACE) might be an explanation for its pharmacological effects. ACE inhibition assay was performed on FXST capsule, 50% ethanol extracts from the four herbals and three selected saponins most abundant in *P. notoginseng* (Ginsenoside Rg1, Ginsenoside Rb1 and Notoginsenoside R1) using a biochemical test. Reversed-phase high-performance liquid chromatography of liberated hippuric acid from the ACE assay was conducted to determine the inhibitory effect. As a result, FXST and extracts from *P. notoginseng* showed a significant and dose-dependent inhibition on ACE activity with the IC_50_ values of 115 μg/ml and 179 μg/ml, respectively. But extracts from the other three herbals and the three selected saponins had no significant effect on ACE inhibition. Compared to other reported plant extracts, FXST could be considered as an effective ACE inhibitor. The inhibition of ACE activity supports the traditional use of FXST on blood circulation and the inhibitory property of FXST is mainly caused by *P. notoginseng*.

## Abbreviations

*FXST capsule:* Fufang Xueshuantong capsule
*ACE:* angiotensin converting enzyme
*RAS:* renin–angiotensin system
*TCM:* traditional Chinese medicine
*HHL:* histidine-l-hippuryl-l-leu-cine-chloride
*HPLC:* high-performance liquid chromatography
*SEM:* standard error of the mean
*IC_50_:* half-maximal (50%) inhibitory concentration (IC) of a substance

## Introduction

The renin–angiotensin system (RAS) plays a key role in the pathogenesis of cardiovascular disease and over-activation of RAS has been considered to be a major causative factor in the development of hypertension.[1] This system is regulated by angiotensin converting enzyme (ACE) and inhibition of ACE is a promising way to control the over-activation of RAS.[2] Several ACE inhibitors, e.g. captopril, enalapril, lisinopril and temocapril are among the most commonly used drugs in the treatment of hypertension and coronary heart disease, as these agents have been proven to effectively reduce the risk of cardiovascular morbidity and mortality.[3] However, all of these drugs produced side effects such as cough, angioneurotic edema and deleterious effects in pregnancy.[4] Natural ACE inhibitors continue to be investigated in different research studies.[5]

Fufang Xueshuantong (FXST) capsule, developed two decades ago according to the meridian theory of traditional Chinese medicine (TCM), has been approved by the State Food and Drug Administration of China for treatment of retinal vein occlusion and stable angina pectoris in 2003 (state medical license No. Z20030017). Many years of clinical application have proved its beneficial effects on diseases associated with blood circulation. However, little is known about the mechanism underlying its effects. FXST is composed of *Panax notoginseng*, *Radix Astragali*, *Radix salvia Miltiorrhizae* and *Radix Scrophulariaceae* in the ratio of 6:2:1:2. Therein, *P. notoginseng* is not only the most abundant, but also the principal herb as far as the theory of TCM is concerned, while the other three herbs are considered as adjuvants that assist the effects or facilitate the delivery of the principal component.[6,7]

In view that ACE over-activation plays an important role in the pathogenesis of cardiovascular disease, as far as we know there are no reports whether the effects of FXST are associated with the inhibition of ACE. The present study aimed to find whether FXST has any effect on ACE activity. Since FXST is composed of four herbal medicines, extracts from the four herbals and three selected saponins (Ginsenoside Rg1, Ginsenoside Rb1 and Notoginsenoside R1) most abundant in *P. notoginseng* were also investigated.

## Materials and methods

### Materials

FXST powder (Batch No. 110817) and herbal extracts were provided by Zhongsheng Pharmaceutical Co. (Guangdong, China). In the manufacture, FXST is the 50% ethanol extraction of *P. notoginseng* (Batch No. 130115 from Yunnan, China), *R. Astragali* (Batch No. 130103 from Gansu, China), *R. Scrophulariaceae* (Batch No. 130110 from Shandong, China) and *R. Scrophulariaceae* (Batch No. 130114 from Hunan, China) in the ratio of 6:2:1:2. *P. notoginseng* was separately extracted and the other three herbals were extracted together, so the herbal extracts were supplied as extracts from *P. notoginseng* (Batch No. 111015) and extracts from the other three herbals (Batch No. 111110). For the experiment, 1 g FXST or herbal extracts powder was solved in 20 mL sterile water and then filtered twice through a standard filter of 0.45 mm in size. The obtained filtrate was considered as 50 mg/ml and was frozen at −20 °C in aliquots until use.

Ginsenoside Rg1 (Batch No. 110703-201027), Ginsenoside Rb1 (Batch No. 110704-200921) and Notoginsenoside R1 (Batch No. 110745-200313) were purchased from the National Institutes for Food and Drug Control (Beijing, China) and dissolved in 50% methanol for the ACE inhibition assay. ACE extracted from rabbit lung (A6778), histidine-l-hippuryl-l-leu-cine-chloride (HHL, H1635) and hippuric acid (112003) were purchased from Sigma-Aldrich (St. Louis, MO, USA), high-performance liquid chromatography (HPLC) grade ethanol and acetic acid were purchased from Burdick & Jackson (Honeywell, USA). Other materials and chemicals were purchased from Beyotime (Shanghai, China). The purity of all chemical reagents was at least of analytical grade.

### ACE inhibition assay

The ACE inhibitory activity of FXST and other samples were evaluated according to the methods of Li et al. [8] with slight modifications. For each assay, a solution of tested sample (20 μl) with 50 μl of HHL solution (5 mM in 50 mM 4-(2-hydroxyethyl)-1-piperazine ethanesulphonic acid (HEPES) buffer containing 300 mM NaCl, pH 8.3) was pre-incubated at 37 °C for five minutes. The reaction was initiated by the addition of 10 μl of ACE solution (100 mU/ml), and the mixture was incubated at 37 °C for 60 minutes. Finally, 100 μl of 1 M HCl was added to stop the reaction. Corresponding volumes of H_2_O or 50% ethanol were used as blank controls to determine the background of ACE-inhibition, while captopril, a known ACE inhibitor, was used as a positive control to ensure the accurate function of the ACE assay. All measurements were performed in triplicate.

After enzymatic reaction, hippuric acid, formed from HHL was quantified with the Ultimate 3000 HPLC system (Dionex, USA). The data were obtained on an Ultimate SB-C18 (15 cm × 4.6 mm, 5 μm) column with the mobile phase composed of 0.1% (v/v) acetic acid in H_2_O/ethanol (75/25, v/v). The flow rate used to achieve a retention time of five minutes was 1 ml/min. The hippuric acid detection was carried out at 228 nm with a DAD-3000 diode array detector. Commercial hippuric acid was used as the standard.

In comparison with the blank control, the % inhibition of ACE was calculated as follows:


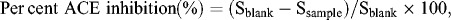


where *S* represents the peak area for hippuric acid. The half-maximal (50%) inhibitory concentration (IC_50_) value was defined as the concentration of inhibitor required to inhibit 50% of the ACE activity under the assayed conditions and determined by regression analysis of ACE inhibition (%) versus log (concentration of test samples).

### Statistical analysis

All data are presented as the mean ± standard error of the mean (SEM). Statistical analyses were carried out using SPSS 16.0. One-way analysis of variance (ANOVA) followed by the Student–Newman–Keuls test were used for comparing the results between groups. *P*-values of less than 0.05 were regarded as statistically significant.

## Results and discussion

To ensure the accurate function of the ACE inhibition assay, captopril, a known ACE inhibitor used to treat hypertension and congestive heart failure, was used as a positive control. The measured IC_50_ for captopril was 1.4 nM (data not shown), which was similar to the measurements shown in most literature ranging from 0.75 to 23 nM.[9]

**Figure 1.**
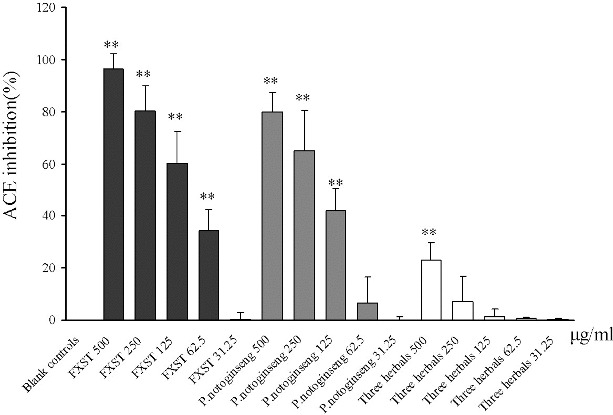
ACE inhibitory activity of Fufang Xueshuantong (FXST) powder and extracts from its component herbals. All the samples were provided by Zhongsheng Pharmaceutical Co. (Guangdong, China). *P. notoginseng* was separately extracted in 50% ethanol and the other three herbals including *R. Astragali*, *R. Salvia* and *R. Scrophulariaceae* were extracted together with the same solvent, and finally the two extracts were mixed to form FXST. The results are the mean ± SEM (*n* = 3), statistical significance is denoted as **, *P* < 0.05, compared to the blank controls.

Compared with the blank controls, FXST showed a concentration responsive ACE inhibition in the dosages 62.5, 125, 250 and 500 μg/ml (Figure 1), giving an IC_50_ value of 115 μg/ml. Compared with other plant extracts that have been investigated on ACE inhibition such as extracts from green tea, blueberry, *Hibiscus sabdariffa*, *Senecio inaequidens* and *Aspilia helianthoides* with the IC_50_ values of 125, 46, 91, 192 and 133 μg/ml, respectively,[10–12] FXST in our study could be thought as an effective ACE inhibitor.

Since FXST is composed of four herbals, the ACE inhibition assay was also applied to the extracts from the component herbals. As a result, extracts from *P. notoginseng* showed a significant inhibition with an IC_50_ value of 179 μg/ml. However, the mixed extracts from *R. Astragali*, *R. Salvia* and *R. Scrophulariaceae* also showed weak effect on ACE activity (Figure 1) and the IC_50_ value could not be obtained solely by *P. notoginseng*. Thus, it could be speculated that *P. notoginseng* played a major role in the ACE inhibitory activity of the FXST formulations. But the IC_50_ value of *P. notoginseng* was higher than that of FXST, suggesting that the complete FXST showed stronger ACE inhibitory activity than *P. notoginseng* alone. The reason might be explained by the synergistic nature of Chinese formulations. As far as the theory of TCM is concerned, the component herbs in a formula usually have complex modes of interactions such as additive, synergistic, restraint and antagonistic. These interactions are considered to be essential for improving their therapeutic potential,[13] although the underlying molecular mechanism is not fully understood.

The principal herbal component in FXST formula, *P. notoginseng*, has been used for hundreds of years in China for its beneficial effect on blood circulation. Extensive chemical studies on this drug have shown that *P. notoginseng* contains a variety of active ingredients including saponins, flavonoids, volatile oil, polysaccharides, alkaloids, amino-glycosides, etc.[14,15] Among these components, saponins are the main bioactive principals and their amount in the raw herb can be as high as 12% on average.[16] For better understanding of the chemical basis in *P. notoginseng* and FXST for ACE inhibition, the three saponins most abundant in *P. notoginseng* were also investigated. As a result, all three saponins exerted weak inhibition on ACE activity (data not shown). Based on these findings, it can be speculated that the high ACE inhibition of *P. notoginseng* is not caused by saponins, but maybe by flavonoids or even polysaccharides, as these components have been described to possess ACE inhibiting activity *in vitro* in other reports.[17,18] Further studies for identification of compounds specifically resulting in ACE inhibition by *P. notoginseng* need to be carried out by its systematic screening.

In recent years, medicinal herbs are being accepted and are increasingly being used by the general population, and many plant extracts have been investigated for their ACE inhibitory properties. Although none of the plant extracts including FXST showed similar IC_50_ values with the chemically synthesized ACEI drugs such as captopril, we cannot say that herbal drugs have no application prospects. In contrast to chemical drugs which are developed to antagonize specific pathological targets or eliminate specific pathological factors, most herbal drugs are regarded as multi-components aimed at multiple targets to treat a totality of different symptoms. This theory of TCM is in line with the trend of modern medicine as using multi-target strategies for treating complicated diseases including many cardiovascular pathologies.[19] Besides the ACE inhibitory properties that are reported here, the component herbals of FXST have been shown to have lipid-lowering, anti-inflammation and anti-oxidation effects,[20–22] which may be all responsible for its beneficial effects on the cardiovascular system.

## Conclusions

FXST exerts significant inhibition on ACE activity, which may be one explanation of its pharmacological effect on the cardiovascular system. FXST formulations are composed of four herbals: *P. notoginseng*, *R. Astragali*, *R. Salvia* and *R. Scrophulariaceae*; *P. notoginseng* plays the major role in ACE inhibition. However, the presence of strong *in vitro* activities does not necessarily imply the *in vivo* results, and further animal and clinical studies are warranted to confirm these results.
